# Unequal voices: examining autism identification and diagnosis disparities for indigenous Mixtec families

**DOI:** 10.3389/fpsyt.2026.1767200

**Published:** 2026-04-13

**Authors:** Paul Luelmo, Fernanda Anahi Castellón

**Affiliations:** 1 Department of Special Education, San Diego State University, San Diego, CA, United States; 2 University of California, Santa Barbara, Santa Barbara, CA, United States

**Keywords:** autism disproportionality, Hispanic families, indigenous populations, language barriers, Latinx, special education

## Abstract

Autism racial/ethnic disproportionality in special education is a significant concern in California and beyond, with White students often overidentified and Latinx and Indigenous (Zapotec/Mixtec) students under-identified. This mixed-methods study investigates the root causes of autism racial/ethnic disproportionality in a California high school district identified as significantly disproportionate for the overidentification of White students with autism. The study was conducted in two stages. First, a Likert-type scale survey (N = 147) was administered to caregivers to examine autism identification and service barriers. In the second stage, three open-response questions within the survey were used to gather qualitative insights from Latinx and Indigenous caregivers. Findings reveal systemic cultural and linguistic barriers contributing to the delayed diagnosis of autism in Latinx and Indigenous students. The qualitative responses further underscore the need for early screening, translation services, and culturally sensitive caregiver support particularly for Indigenous, Mixtec families.

## Introduction

Autism is a complex developmental condition that has gained significant attention due to its increasing prevalence and the disparities in identification, diagnosis, and service access across different demographic groups in the USA (1). While racial/ethnic disparities in autism identification and services access are well documented for various racial/ethnic groups (1), and more recently addressed in order to lessen disparities (2). However, to our knowledge, little is known about Indigenous children and autism identification in the United States (US) (Kristofik, A., & Demps, K. (3).

Likewise, racial/ethnic disproportionality in special education, where certain racial and ethnic groups are overrepresented or underrepresented, has been well documented in the literature (4–6). In the case of autism, White students are often more likely to receive an early autism diagnosis, while Hispanic/Latine students face significant under identification and underservice (7). In particular, Hispanic/Latine students are more likely to be identified with learning disability and intellectual disability than their white peers (8). Indigenous students from Latin American backgrounds, those whose language and culture might differ from mainstream Latines, are often categorized as “Latine/Hispanic” (9, 10). In this article we use the term Hispanic, and Latine interchangeably. These disparities in autism identification are not merely the result of diagnostic processes but are influenced by broader systemic issues, such as language barriers, cultural differences, and the inequitable availability of early screening and intervention services (1, 11). The overrepresentation of minority students in special education was first brought to national attention by Dunn (12) and has since become a focus of federal and state monitoring efforts, culminating in the inclusion of specific provisions addressing disproportionality in the Individuals with Disabilities Education Act (IDEA) of 2004.While Hispanic refers to students from Latin American background in state and federal demographic constructs, the gender-neutral “Latine” is preferred to refer to this community, more aligned with people from Spanish-speaking countries in Latin America.

As a result of IDEA’s mandate, states and local educational agencies (LEAs) are required to continuously monitor the potential disproportionate representation of ethnic and minority students (7, 13). An integrated synthesis of disproportionate representation of minority students in special education by Cruz & Rodi (7) found that risk ratios were the second most common type of statistical measure of disproportionality. This measure is after the risk index (RI) the probability that a student from a given racial/ethnic group is identified for special education) (7). Risk ratios are a strong tool for comparing the likelihood that students from a specific racial or ethnic group are placed in special education compared to students from all other groups, helping to identify patterns of disproportionate representation. Risk ratios are powerful because they compare the relative likelihood of special education placement for one racial or ethnic group against all other students, while accounting for differences in group size. For example, if 15% of Black students and 10% of all other students are placed in special education, the risk ratio is 1.5, meaning Black students are 50% more likely to be placed compared to their peers.

### Linguistic and cultural diversity within indigenous Mesoamerican communities

Discussions of autism disproportionality among Latine populations often hide significant linguistic and cultural heterogeneity within Indigenous Mesoamerican communities. Mesoamerica includes multiple major language families—including Otomanguean, Mayan, and Mixe-Zoquean—comprising well over 250 distinct Indigenous languages (14; Suárez, 1983). Contrary to common belief, these languages are not dialects of Spanish, nor are they mutually intelligible across families. Instead, these languages represent historically and structurally distinct linguistic systems with unique phonologies, morphologies, and syntactic structures.

Within this diversity, “Mixtec” refers not to a single language but to a branch of the Otomanguean language family comprising approximately 50–55 distinct languages, many of which exhibit limited mutual intelligibility (15, 16). Likewise, “Zapotec” constitutes a family of over 50 distinct languages associated with specific municipalities and communities, often differing substantially in phonological and lexical systems (14, 17). Grouping these languages under a single label is comparable to combining Romance or Germanic languages into one analytic category. The use of broad ethnic labels such as “Latinx” “Latine” or “Hispanic” further hides this variability. Many Indigenous families from Oaxaca and surrounding regions may be considered Hispanic while primarily speaking an Indigenous language at home. Prior research has documented how schooling institutions frequently ignore or misclassify Indigenous Latino languages, rendering them invisible within bilingual education and special education systems (18). Such erasures may contribute to structural barriers in disability identification processes that rely heavily on Spanish-language communication and written documentation.

In the present study, we focus specifically on caregivers who reported speaking Mixtec or Zapotec language varieties. We acknowledge the limitation in this sample and how it represents only a subset of the broader Otomanguean-language families and does not capture the full linguistic diversity of Indigenous Mesoamerica. When referring to “Mixtec/Zapotec-speaking caregivers,” we do so for analytic clarity while recognizing the internal diversity and variability of these language families. Better precision in describing linguistic subgroups is essential for interpreting findings related to language access, interpreter availability, and diagnostic pathways and a future are of research.

### The present study

A High School District in California’s Central Coast served as the study site. The district’s demographic composition, predominantly Hispanic/Latine with a significant Indigenous Mixtec/Zapotec population, provided a unique context to examine autism disproportionality in schools with a particular focus on Latine and the demographic subgroup of Indigenous communities, specifically Mixtec and Zapotec. The district had been identified for significant disproportionality by the California Department of Education, with White students overrepresented in autism identification. However, previous research (19) found that a contributing factor to the over-representation of White students was the under-representation of Latine students, particularly those from Indigenous backgrounds.

### Ethical considerations

The study adhered to ethical research standards, ensuring informed consent, confidentiality, and cultural sensitivity. Special efforts were made to provide language support for Indigenous (i.e., Mixtec and Zapotec) participants to facilitate meaningful engagement. Institutional Review Board approval was obtained from a University in Southern California (IRB-25-0557).

## Methods

The school district in the study was identified by the California Department of Education (CDE) as significantly disproportionate for this overrepresentation of White students in the special education category of autism for several years. The district has been involved in the State-mandated system improvement plan and implementation (CCEIS). As part of this State mandated process, the district conducted several studies to identify the root cause of the disproportionality (20, 21). The State Performance Plan- Technical Assistance facilitators conducted multiple studies over the years. This article presents the findings of a culminating study aimed at finding the root cause(s) of disproportionality in autism for White students, with a particular interest in the identification, diagnosis, and perceptions of caregivers of Autistic Indigenous students.

### Research design

The mixed-methods approach was selected to integrate numerical trends with open-ended narrative accounts by caregivers. This design allowed for a comprehensive analysis of how cultural, linguistic, and systemic factors influence autism diagnosis and service access for Mixtec families.

### Research questions

**Research Question 1 (RQ1):** In a district with documented autism disproportionality, to what extent are children of non-English-speaking caregivers-particularly those from Indigenous Mixtec language backgrounds- less likely to receive a medical autism diagnosis compared to children of English-speaking, or Spanish-speaking non-Indigenous caregivers?

**Research Question 2 (RQ2):** How do caregivers’ perspectives on identification, services, and school support differ across demographic groups?

#### Participants

Participants were parents of children with an IEP with autism and/or other developmental disabilities category in the district. The total sample included 147 caregivers. Of these, 37 identified as Indigenous (Mixtec speaking), 48 as non-Indigenous Spanish-speaking, and 49 as non-Indigenous English-speaking. One caregiver identified as Indigenous (English-speaking). An additional 12 caregivers were categorized as other or combined.

#### Data collection

The survey was administered online to ensure accessibility for participants across the district. It was available in both English and Spanish. To increase participation among linguistically diverse families, the district distributed the survey through trusted community channels, including a district community liaison who directly shared the survey with parents of children with Individualized Education Programs (IEPs). For families who speak Mixtec, a Spanish version of the survey was orally translated into Mixtec by a Mixtec-speaking parent liaison employed by the district. Given that Mixtec is traditionally an oral language without a standardized written form, participant responses in Mixtec and Zapotec were documented in Spanish to ensure accurate recording and analysis.

#### Mixed methods design and integration

This study utilized an exploratory mixed-methods design to integrate quantitative patterns of diagnostic disparities with qualitative caregiver’s points of view (22). This mixed-methods approach was used to examine not only whether disparities in medical diagnosis and school-based identification of autistic students manifested across linguistic and cultural groups but also investigate how caregivers understood and experienced these. Thus, in the final analysis we integrate numerical findings with narrative accounts of caregivers captured in the open-responses of the survey. Moreover, we conclude with a more comprehensive analysis of how cultural, linguistic, ethnic and systemic factors influence autism identification, and importantly, access to services for Indigenous Mixtec/Zapotec families.

Specifically, the quantitative phase of the analysis addressed RQ1, by examining the likelihood of receiving a medical diagnosis of autism across caregiver language groups in a district with a documented racial/ethnic disproportionality of autism. Moreover, we compared children of English-speaking caregivers, Spanish speaking non-indigenous caregivers, and Indigenous Mixtec/Zapotec-speaking caregivers.

To answer RQ2, we employed a qualitative phase by analyzing caregivers’ perspectives on identification, service access and school support. The open-ended responses in the survey, as well as the short oral interviews, gave us contextual data into how language access, cultural understanding of disability, institutional trust, and communication with school staff shaped the experience of caregivers. Hence, the qualitative analysis deepen interpretation of the quantitively analysis.

Integration of findings happened during the interpretation phase through narrative weaving. Quantitative findings identified disparities for Mixtec/Zapotec families along with qualitative themes that shed light into potential mechanisms behind those disparities. For instance, patterns of delayed diagnosis or no diagnosis was interpreted in relation to caregivers describing language barriers, limited access to culturally responsive information, and heavy reliance in school-based identification. The integration of findings in this approach strengthens this study’s focus on equity, by centering both statistical patterns and participants lived experiences.

#### Quantitative stage

The quantitative phase involved a caregiver survey designed to capture demographic characteristics and experiences with autism diagnosis and the Individualized Education Program (IEP) process. The survey was adapted from previous studies addressing gaps in identification and service access for ethnic/racial minoritized communities they survey was validated using a community-based approach, and validated in Spanish, English and Korean (1).

#### Qualitative stage

In a previous study (Luelmo, et al., 2021) it became evident that many caregivers identified as Latine were also part of a specific Latine population- Indigenous Mixtec and Zapotec. Hence, the decision to dive deeper into cultural and linguistic differences that may have impacted how parents received autism identification and services in school. Differentiating the Latine subgroups (Latine English speaking, Latinx Spanish speaking, and Latinx Zapotec or Mixtec-speaking) became a central question in the study. As part of the online survey data collection method, participants were also asked to answer a 3-item open-ended response. Participants who responded by phone (i.e., Mixtec/Zapotec) responded to a short oral interview, a trusted school-based community liaison and interpreter conducted the interviews (Lasting 15–20 min).

Importantly, the qualitative stage, was guided by reflexive thematic analysis (23), We selected this approach to allow for systematic identification of patterns across caregivers groups while remaining attentive to the sociocultural and linguistic contexts of the participants’ lived experiences.

### Coding process

The first author conducted the initial phase of open-coding, reading all responses multiple times to become familiar with the dataset. Then codes were generated inductively, to capture patterns in language access, diagnostic pathways, home-school communication, perceptions of facilitators or helpers, and systemic barriers. Analytic memos were written and maintained by the first authors during the coding process and document emerging interpretations and reflexive considerations, specifically regarding positionality and cultural interpretation.

Following the initial coding phase, codes were grouped into themes within and across demographic groups. Particular attention was given to differences between Spanish-speaking non-indigenous caregivers and Mixtec/Zapotec speaking indigenous caregivers, this was important given the study focus on differentiation within Latine populations. Then, feedback was solicited from the second author, who conducted an analysis of the thematic analysis and its interpretation.

### Use of generative- AI

To improve analytic transparency and triangulation, the dataset was de-identified and uploaded to an institutional and secure GenAI workspace. The AI was asked to conduct a thematic analysis using the research questions and identifying the four demographic groups for analytic purposes. The AI-generated themes were compared to the researcher generated codes and themes. Remarkably, both analytic approaches resulted in strikingly similar themes in regard to language barriers, trust, and reliance on school-based identification of autism. AI output weas not used as substitute for human analysis but used a supplementary analytic tool to support validation of findings and to identify salient excerpts included in this manuscript. Final thematic analysis, interpretation and reporting remained the responsibility of the authors.

### Positionality statement

The authors approach this study with a commitment to educational equity and the reduction of disproportionality in special education. The first author is a *transfronterizo* scholar whose work centers culturally and linguistically diverse communities navigating educational systems across language boundaries. The second author has experience in community-engaged research with multilingual families and school–family partnerships in under-resourced contexts.

Although neither author identifies as Mixtec or Zapotec, we have longstanding professional relationships with Indigenous-serving communities and collaborated with district cultural and linguistic liaisons throughout data collection and interpretation. We engaged in reflexive memoing (i.e., recording, tracking, and exploring ideas/insights and relationships in qualitative data) and peer consultation to remain attentive to issues of power, translation, and cultural interpretation, and we intentionally centered caregiver voices through direct quotation and linguistic disaggregation.

## Measures

### Demographics

Demographic data was collected from caregivers completing the study and aggregated by analytic grouping. **Table 1** shows all participants’ demographics by analytic group.

**Table 1:** Demographics by analytic groupings.

Caregiver group	N=147
Indigenous (Mixtec speaking)	*37*
Non-Indigenous (Spanish)	*48*
Non-Indigenous (English)	*49*
Indigenous (English speaking)	*1*
Other/combined	*12*

#### Caregiver autism identification facilitators and barriers

Researcher- adapted previously used qualitative measure (1), into a quantitative tool to gather insights into autism identification, barriers, and facilitators (e.g., Did you child have an autism diagnosis, prior to receiving an IEP in school)?

#### Open-ended survey questions

Researched-developed items based on intimate knowledge of the community and the disproportionality in the district to gather additional input into specific root causes of autism under identification for Latine and Indigenous population (e.g., Who was helpful in identifying your child for autism)?.The survey captured caregiver-reported barriers to autism identification/diagnosis and service access (Stahmer et al, 2019).

### Data analysis

Descriptive and inferential statistical methods were used to examine differences across four caregiver groups reflecting key demographic and linguistic characteristics. These groups were derived from combinations of Hispanic/non-Hispanic identity and English/Spanish/Mixtec language use. Specifically, the analytic groups were: English-speaking non-Hispanic caregivers, English-speaking Indigenous caregivers, Spanish-speaking Hispanic caregivers, and Mixtec-speaking Hispanic caregivers. The first author conducted the initial analyses, organizing and tabulating results according to the research questions. To validate these findings, an institutional GenAI workspace was used to independently address Research Question 1 using the original dataset. The GenAI produced the same substantive conclusions as the research team and additionally confirmed the results by applying a Fisher’s Exact Test, which was found to be highly significant. Fisher’s Exact Test is a nonparametric statistical procedure used to compute the exact probability of observing the distribution of counts under the null hypothesis of no association. It is therefore appropriate for small sample sizes or sparse data. In our analysis, Fisher’s Exact Test was applied to assess whether the observed differences between caregiver demographic groups (e.g., English-speaking non-Hispanic, Spanish-speaking Hispanic, Mixtec-speaking Hispanic) were statistically significant. The test yielded a highly significant result, indicating that the variation across groups was unlikely due to chance.

### Qualitative data analysis

As part of the online survey, participants responded to three open-ended questions. For participants who completed the survey by phone—primarily Mixtec-speaking caregivers—responses were collected orally through a school community liaison and interpreter. After data cleaning, all open-response data were tabulated in a spreadsheet and organized by the caregiver demographic group: English-speaking non-Hispanic, English-speaking Indigenous, Spanish-speaking Hispanic, and Mixtec-speaking Hispanic. Data were anonymized, and only de-identified excerpts and group classifications were retained for analysis. The first author conducted an initial open-coding process to identify themes by group, noting early patterns such as pronounced language barriers for Mixtec-speaking caregivers. To validate and complement the coding process, the spreadsheet was uploaded to an institutional GenAI workspace. The prompt requested a thematic analysis disaggregated by the same four demographic groups. Results from the researcher-generated coding and GenAI-generated analysis were then compared. The two approaches yielded highly similar themes, and the representative quotes included in the results were those identified by GenAI as the most illustrative of each theme.

## Results

RQ2: to what extent are children of non-English-speaking caregivers—particularly those from Indigenous language backgrounds—less likely to receive a medical autism diagnosis compared to children of English-speaking, non-Indigenous caregivers.

In stage 1 (quantitative survey data), researchers tabulated the under-identification of Latinx students with autism and revealed that non-English-speaking families, particularly those from Indigenous Mixtec backgrounds, encountered significant difficulties in navigating the IEP process. It also became evident that a significant number of caregivers identified as Hispanic/Latinx/Latine were specifically from Indigenous communities in Oaxaca in Central México, belonging to the Zapotec/Mixtec family of pre-Hispanic cultures and languages. Indigenous families frequently reported a lack of adequate translation services and culturally competent support, which delayed autism diagnosis and intervention services. This finding corroborates research by Zuckerman et al. (24), who identified language as a primary barrier to timely autism diagnosis in Latine families. Furthermore, Zarate et al. (25) highlighted the unique challenges faced by Indigenous families, including limited resources in Indigenous languages and a general lack of awareness about autism within the Indigenous community.

Children of Indigenous (non-English speaking) caregivers are significantly less likely (RR = 0.50) to receive an autism diagnosis before receiving an IEP in school than English-speaking non-Indigenous caregivers. Importantly, roughly 30% of Indigenous (Spanish) respondents reported a prior diagnosis of the IEP (See **Table 2**). By contrast, 60% of English-speaking caregivers and 86% of non-Indigenous Spanish-speaking caregivers reported receiving an autism diagnosis before receiving an IEP in schools. This result is statistically significant and consistent with the thematic analysis that language *and* culture play a role in a timely diagnosis of autism. See **Tables 3**, **4**.

**Table 2:** Autism barriers and facilitators.

Group	N	Parent education (higher ed)	Diagnosis before IEP	Most common barrier	School staff helpfulness*
English-speaking	49	26%	67%	*No barriers*	—
Indigenous (Mixtec/Zapotec)	37	0%	39%	*El lenguaje (Language)*	89%
Spanish-speaking (non-Indigenous)	61	0%	89%	*El lenguaje (Language)*	96%

*Participants rated their agreement (Strongly Agree to Strongly Disagree) with the statement: “School staff were helpful in facilitating my child’s IEP.”

**Table 3:** Caregiver group responses by language and indigenous identity.

Caregiver group	Yes	No	Unsure	*n*	Yes rate (%)
Indigenous (Mixtec speaking)	11	17	9	*37*	29.7
Non-Indigenous (Spanish)	42	5	2	*49*	85.7
Non-Indigenous (English)	21	11	3	*35*	60.0
Indigenous (English speaking)	1	0	0	*1*	100.0¹

Yes Rate (%) represents the percentage of “Yes” responses within each caregiver group to the survey item “Did you child have an autism diagnosis, prior to receiving an IEP in school)?. A total of 25 participants left the diagnosis item blank.

¹*n* = 1 (interpretation not significant due to low n).

**Table 4:** Interpretation of patterns in medical diagnosis before IEP.

Contrast	Observation	Relative likelihood (RR)
Indigenous (Spanish) *vs*. Non-Indigenous (Spanish)	Far less likely to have a medical diagnosis before IEP	RR ≈ 0.35 (−65%)
Indigenous (Spanish) *vs*. Non-Indigenous (English)	Also, substantially lower (29.7% *vs*. 60%)	RR ≈ 0.50
Indigenous (Spanish) *vs*. Non-Indigenous (any language)	Consistently below both comparison groups	—

Fisher’s exact test (Indigenous *vs*. Non-Indigenous Spanish): *p* < 1.3 × 10^-7^ (highly significant).

### Demographics

A total of 147 caregivers participated in the current study across four demographic groups (see **Table 1** for a detailed breakdown by language and Indigenous identity). The total sample included 147 caregivers. Of these, 37 identified as Indigenous (Mixtec speaking), 48 as non-Indigenous Spanish-speaking, and 49 as non-Indigenous English-speaking. One caregiver identified as Indigenous (English-speaking). An additional 12 caregivers were categorized as other or combined. Notable disparities emerged in educational attainment and diagnostic timing across groups. Among English-speaking non-Hispanic caregivers, 26% had completed higher education, compared to 0% among both Indigenous Mixtec-speaking and Spanish-speaking Hispanic caregivers. The timing of autism diagnosis relative to receiving an Individualized Education Program (IEP) varied substantially by group. English-speaking non-Hispanic caregivers reported that 67% of their children received an autism diagnosis before IEP placement, while Spanish-speaking Hispanic caregivers reported the highest rate at 89%. In stark contrast, only 39% of Indigenous Mixtec-speaking caregivers reported receiving a diagnosis before their child’s IEP. Language barriers emerged as the most commonly reported obstacle for both Spanish-speaking (96% reported school staff helpfulness despite barriers) and Mixtec-speaking families (89% reported school staff helpfulness), while English-speaking caregivers predominantly reported no barriers to accessing services. These figures reflect considerable disparities in access to early diagnosis and intervention, particularly for Indigenous language-speaking families navigating systems without adequate linguistic and cultural support.

### Thematic analysis

The application of qualitative coding and thematic analysis to the open-ended survey responses resulted in three primary themes: 1) Access to early diagnosis and services, 2) Language barriers and system navigation challenges, and 3) Heavy reliance on institutional support systems. See **Table 2** for group-specific patterns in diagnosis timing, barriers, and support experiences.

#### Access to early diagnosis and services

English-speaking non-Hispanic caregivers in the current study described their experiences accessing early diagnosis and intervention services as relatively straightforward, with many receiving timely referrals from healthcare providers and educators. From the beginning of their child’s autism journey, these caregivers benefited from proactive identification by professionals who recognized developmental concerns early. One English-speaking caregiver shared the efficiency of their diagnostic process: “It was quick, and intervention even quicker. He was getting services for vision issues, and he was referred right away to be evaluated for autism.” The accessibility of early services was further emphasized by another caregiver who described receiving in-home assessments: “His doctor was helpful, along with Santa Barbara County Education. They tested my son in my home.” For these families, the system’s responsiveness facilitated early intervention, though some still encountered difficulties. One caregiver reflected, “Getting him tested was difficult. Had him privately assessed as well to make sure the diagnosis was somewhat accurate. Navigating resources was challenging.”

Caregivers also faced educational barriers related to professional awareness. Some received strong teacher support, with one caregiver noting, “*My son’s preschool teacher was the one who first noticed something and helped*.” Others struggled with educators who lacked awareness or training in autism diagnosis: “*At first, it was staff. At that point in time, educators were not proficient with the diagnosis of autism. So any complaint or issues the child would have were ignored with simple explanations like ‘well, it can be behavior or attention-seeking*.’”

Spanish-speaking Hispanic caregivers relied heavily on the school system for autism identification and accessing services rather than pursuing independent medical referrals. These families frequently learned of their child’s needs through teacher observations. One caregiver explained, “*The teachers realized that my daughter needed help because she had learning difficultie*s.” Another noted the central role of school staff in initiating evaluations: “T*he school staff helped us evaluate my child*.” This dependence on educational institutions for initial identification often meant delays in accessing services, as families waited for school personnel to notice developmental differences.

Mixtec-speaking Hispanic caregivers faced the most severe barriers to early diagnosis and services. Several caregivers indicated they had no framework for understanding autism or disabilities before arriving in the United States. One Mixtec-speaking caregiver reflected on this knowledge gap: *“I realized that my son had a disability in Mexico, but he did not receive support. He only started receiving support when he arrived in the United States*.” Another caregiver described their complete unfamiliarity with special education: “*I had no idea what an IEP was before the school staff explained it to me*.” The combination of linguistic barriers and limited prior exposure to disability services meant that Mixtec-speaking families were entirely dependent on institutional actors to both identify their child’s needs and guide them through the intervention process.

### Language barriers and system navigation challenges

Although English-speaking non-Hispanic caregivers generally navigated the special education system with relative ease, they encountered challenges related to educator awareness and diagnostic accuracy. Several caregivers described taking independent advocacy steps to ensure their children received appropriate evaluations. One caregiver, despite being an educator themselves, shared: “*Although I’m a teacher myself and saw the red flags, it was difficult to get him tested and diagnosed through the school system*.” Another caregiver pursued a private assessment: “*I had him privately assessed because I wanted to make sure the diagnosis was accurate.*” One caregiver captured the emotional weight of navigating the system: “*It was terrifying. Very little assistance, no one knew what to do to help. The school psychologist had never seen a student test so high for emotional disturbance.*”

For Spanish-speaking Hispanic caregivers, language emerged as the primary and most persistent barrier throughout the special education process. Caregivers repeatedly emphasized how linguistic differences impeded their understanding of complex educational procedures. One Spanish-speaking caregiver articulated this challenge directly: “*More than anything, the language barrier. There is a lot that sometimes we don’t understand.”* Another expressed the difficulty of comprehension: “*The language. I try to understand, but it is difficult for me*.” The pace of communication compounded these challenges, as one caregiver noted: “*I don’t* sp*eak any English, and they* sp*eak very fast.*”

Spanish-speaking caregivers also struggled to understand the IEP process and their rights. Even when translation services were provided, caregivers questioned their adequacy: “*The school staff did help us, but the language was very difficult for me. I didn’t understand those things about the IEP.*” Another caregiver shared, “*Sometimes I didn’t understand what they were telling me, and the translators did help, but I feel like they didn’t know how to explain well in Spanish.*” The language barrier extended beyond simple translation to encompass understanding specialized educational terminology and procedural rights.

Mixtec-speaking Hispanic caregivers experienced the most profound language barriers, as they navigated systems with virtually no linguistic or cultural accommodation. Unlike Spanish-speaking families who could at least access Spanish-language interpreters, Mixtec-speaking caregivers had no access to interpretation in their native language. One caregiver described the isolation of this experience: “*The language barrier was the hardest. I didn’t understand anything at first. There was no one who could explain to me in Mixtec.”* The absence of Mixtec language support meant these families operated in a state of near-total dependence on institutional guidance. As one caregiver simply stated: *“Sometimes we didn’t know what to do, but the school staff helped us a lot.*”

#### Heavy reliance on institutional support systems

English-speaking non-Hispanic caregivers demonstrated the capacity for independent advocacy, pursuing private assessments and researching interventions on their own. Many caregivers mentioned taking independent steps to confirm school diagnoses or access additional services. While some praised educators who provided early support, their ability to supplement or challenge school services through private resources reflected a degree of autonomy within the system. The variability in teacher support meant that these caregivers often needed to advocate actively for their children’s needs.

Spanish-speaking Hispanic caregivers demonstrated marked dependence on school-based systems for both identification and ongoing support. The reliance on educators and school psychologists was evident throughout their narratives, as families looked to institutional actors to recognize their children’s needs and initiate appropriate interventions. While caregivers expressed appreciation for school staff assistance—with 96% reporting that staff were helpful—this high satisfaction rate must be understood within the context of limited alternatives. Unlike their English-speaking counterparts, who could supplement school services through private assessments, Spanish-speaking families operated primarily within the boundaries of what schools offered.

Mixtec-speaking Hispanic caregivers exhibited the highest degree of institutional dependence, relying almost exclusively on doctors and school staff to guide them through every aspect of the diagnostic and educational process. Medical professionals played a particularly central role in helping these families understand their children’s conditions. One caregiver described how healthcare providers explained their child’s diagnosis: “*The doctors and nurses helped us with the diagnosis. They said that he would not be like other normal children. He has many health problems.”* School staff similarly served as essential guides through the special education system: *“The* sp*ecial education teachers helped us, and they helped my son in his classes.”* The 89% rate of reported school staff helpfulness among Mixtec-speaking caregivers reflects both genuine institutional support and the absence of alternative support structures. Without access to interpretation services in Mixtec or independent advocacy resources, these families had no choice but to place complete trust in the institutional actors who represented their only connection to services for their children.

## Discussion

The study provides strong evidence that Indigenous families—despite targeted outreach conducted in Mixtec and Spanish—experience significantly lower rates of early medical diagnoses of autism (i.e., before receiving an IEP in school). These disparities persist even when survey conditions and recruitment contexts are comparable. This pattern indicates that the barriers are likely structural and linguistic rather than artifacts of sampling (Luelmo, et al., 2022; Luelmo et al., 2024). Notably, Spanish-speaking non-Indigenous caregivers closely resemble English-speaking caregivers in their rates of early diagnosis once basic language access barriers are reduced. In this sample, Spanish-speaking non-Indigenous caregivers reported an early diagnosis rate of 86%, compared with 60% among English-speaking caregivers. This finding suggests that language alone does not fully account for the diagnostic disparities experienced by Indigenous families. Instead, the results point to a more complex interplay among language, Indigenous identity, and system-navigation challenges that collectively contribute to persistent inequities in autism identification.

A critical factor is the limited availability of qualified interpreters for Indigenous languages such as Mixteco and Zapotec—an issue documented both in the U.S. and internationally. Additionally, cultural perceptions of disability, historical mistrust of Western institutions (26, 27), and difficulties navigating U.S. healthcare and educational systems may further delay initial medical visits, referrals, and timely autism diagnoses for Indigenous families.

English-speaking parents were more proactive in seeking private assessments and advocating for their children but still faced teacher resistance and systemic delays. While recent research has focused on barriers and facilitators for autism identification for Latine families (28), closing the diagnosis gap that previously existed, this study sheds light on the often-confounded group of Indigenous children. Importantly, Spanish-speaking parents relied primarily on the school system but faced significant language barriers and lacked knowledge of the IEP process. Moreover, Mixtec-speaking parents encountered the greatest challenges due to language barriers and a general lack of prior knowledge about autism, disabilities, and special education services.

These findings highlight the need for increased linguistic and cultural support, particularly for Indigenous-language speakers, to ensure equitable access to autism diagnosis and special education services. These findings highlight the need for increased linguistic and cultural support, particularly for Indigenous-language speakers, to ensure equitable access to autism diagnosis and special education services. This methodology highlights the systematic approach taken to examine autism disproportionality through robust mixed-methods research.

The findings from both studies highlight systemic barriers that perpetuate autism disproportionality in special education, particularly for Latine and Indigenous populations in California. These results align with a growing body of literature emphasizing the intersection of cultural, linguistic, and systemic factors in creating disparities in autism identification and access to services (1, 7). The challenges faced by these populations reflect broader inequities in the educational and healthcare systems that disproportionately disadvantage underserved communities.

Importantly, the qualitative stage of the study provided qualitative insights into the lived experiences of caregivers, revealing cultural stigmas and misunderstandings about autism as significant barriers to accessing care. Many Mixtec-speaking families needed foundational knowledge about autism and its symptoms, which compounded delays in seeking a diagnosis. These findings are consistent with Dababnah et al. (29), who found that cultural attitudes and stigma frequently deter Latinx families from pursuing developmental evaluations for their children. The qualitative results also echo Gomez et al. (30), who emphasized the critical need for culturally relevant and accessible autism services tailored to the needs of non-English-speaking and culturally diverse families.

Both studies underscore the importance of culturally responsive interventions to address autism disproportionality. Research indicates that improving access to early autism screening and diagnosis for underserved populations requires systemic reforms that include enhanced translation services and culturally sensitive training for educators and service providers (1, 31). Additionally, resources designed specifically for Indigenous families, including those in Mixtec-speaking communities, are essential for reducing the significant disparities identified in this study. This aligns with the recommendations of Zarate et al. (25), who advocate for creating tailored educational materials and caregiver support programs in Indigenous languages.

The findings further suggest that effective solutions must go beyond addressing language barriers. For example, caregiver education initiatives that reduce stigma and increase awareness about autism can empower families to advocate for timely diagnoses and services (24). Educators and professionals working with Latinx and Indigenous families must be equipped with the tools and training necessary to understand and respect the cultural contexts in which these families operate (32, 33).

### Limitations

This cross-sectional study reflects associations rather than causal relationships. Subgroup sizes were uneven, and the combination of Mixtec and Zapotec-speaking caregivers for quantitative analyses may mask subgroup-specific differences. Data relied on caregiver self-report without independent clinical verification, and the survey instrument was developed for community-based purposes rather than as a validated scale. Additionally, because some responses were translated from Mixtec or Zapotec into Spanish and then English, some nuance may have been lost in translation. Future research should include larger Indigenous-language samples and longitudinal designs.

## Data Availability

The raw data supporting the conclusions of this article will be made available by the authors, without undue reservation.
